# Exercise capacity of adolescent girls with idiopathic scoliosis; analyzed in 6 Minute Walking Test (6MWT), with and without Chêneau's brace - pilot studies

**DOI:** 10.1186/1748-7161-8-S1-O18

**Published:** 2013-06-03

**Authors:** J Pajak, J Durmala, J Bugala-Szpak

**Affiliations:** 1 Department of Medical Rehabilitation, Medical University of Silesia, Katowice, Poland

## Aim

Establishing if Chêneau's brace decreases exercise capacity in adolescent patients with idiopathic scoliosis.

## Methods

This is a prospective, randomized study. The research was done in 22 girls (mean age 15±1.7 y.) with DM idiopathic scoliosis (mean Cobb angle - 33 degrees). These patients were qualified to conservative treatment using Chêneau's brace. Every studied person was acquainted with the technique of carrying out 6MWT, and realized the tentative test before proper exercise tests in the aim of the elimination of "the learning's effect". Tests were executed twice, in the brace and without the brace. The order or carrying out each test was random, so that half of the patients executed their first exercise test in the brace and the other half took their first test without the brace.(two various balls were used for the drawing).

## Results

There was a statistically significant longer distance for the walking test, by patients without Chêneau's brace, in comparison to these same patients with Chêneau's brace. (676.2±61.26m vs. 631.8±78.31m) A positive correlation (non significant) was observed among the length of the distance in 6MWT and the size of kyphosis.

## Conclusions

This initial study showed;

1. Chêneau’s brace essentially diminishes exercise capacity for patients with idiopathic adolescent scoliosis.

2. A higher value of the kyphosis angle can reduce the negative influence of Chêneau's brace on exercise capacity.
